# Natural history of lower urinary tract symptoms and voiding parameters in ageing men with 5-year follow-up—the concord health and ageing in men project (CHAMP)

**DOI:** 10.1093/ageing/afaf078

**Published:** 2025-04-06

**Authors:** Giovanni Losco, Lewis Chan, Rachel Esler, Vasi Naganathan, Fiona Blyth

**Affiliations:** Department of Surgery, University of Otago Christchurch, Canterbury, New Zealand; Department of Urology, Health New Zealand Te Whatu Ora Waitaha Canterbury, Christchurch, Canterbury, New Zealand; Department of Urology, Concord Repatriation General Hospital, Concord, NSW, Australia; Department of Urology, Royal Brisbane and Women's Hospital, Herston, QLD, Australia; Centre for Education and Research on Ageing, Concord Repatriation General Hospital, Concord, NSW, Australia; Pain Management Research Institute, University of Sydney, Sydney, NSW, Australia

**Keywords:** bladder, urinary, bladder, ageing, quality of life, older people

## Abstract

**Background:**

Natural history of voiding parameters with age is poorly understood. We aim to understand both subjective and objective lower urinary tract parameters in older men over 5 years.

**Methods:**

The Concord Health and Ageing in Men Project is a prospective cohort study of older men, involving 1705 men aged 70 years and over living in Sydney, Australia. Men were assessed at 0, 2 and 5 years. Demographic information, medical history, International Prostate Symptom Score (IPSS), flow rate and post-void volume were collected at three timepoints.

**Results:**

A total of 1705 men aged 70–97 years participated. At 2 and 5 year follow-up, 1367 and 940 men presented for assessment. Mean IPSS was 7.35 at baseline, 6.96 at 2 years (*P* = .9) and 7.18 at 5 years (*P* = .30). Mean flow rate at baseline was 15.0 ml/s, 14.6 ml/s at 2 years (*P* = .001) and 15.3 ml/s at 5 years (*P* = .42). Adjusting for age at baseline, the change in flow over 5 years was not significant (*P* = .93). Mean post-void residual was 72.4 ml at baseline, 84.0 ml at 2 years (*P* = .003) and 93.2 ml at 5 years (*P* = .001). Men with residual volume >200 ml at baseline had no significant change in residual over 5 years (*P* = .51).

**Conclusions:**

Urinary symptoms and voiding parameters remain stable over 5 years. Men with elevated post-void volume did not deteriorate significantly. Conservative management of lower urinary tract symptoms appears a reasonable strategy in older men.

## Key Points

- Natural history of voiding parameters with age is poorly understood.
- Urinary symptoms and voiding parameters remain stable over 5 years.
- Men with elevated post-void volume did not deteriorate significantly.
- Conservative management of lower urinary tract symptoms appears a reasonable strategy in older men.

## Introduction

Lower urinary tract symptoms (LUTS) are common in older men. They are a source of frequent visits to health care providers, general practitioners, urologists and geriatric medicine specialists [1–3]. LUTS have an adverse effect on quality of life and result in increased costs in men living in the community and residential care facilities [4–6]. Medications and surgery for LUTS are a significant burden on health resources [7]. There have been several studies that have followed the incidence and prevalence of LUTS through time (Baltimore Longitudinal Study of Ageing (BLSA) [8] and the Olmsted County Study of Urinary Symptoms and Health Status [9]). Many of these studies have described the subjective burden in terms of the International Prostate Symptom Score (IPSS), operative rate and urinary flow rate [10].

Symptoms are the major reason for most patients to seek medical attention and consequently a number of symptom scores have been designed in search of an objective assessment. The most widely used is currently the IPSS which is generally accepted as a reliable and valid instrument to measure severity and progression over time [11]. Besides these subjective parameters, changes in objective parameters are used to evaluate disease progression and treatment success. Urodynamic pressure-flow analysis is the reference standard but its role in the diagnostic armamentarium is not without controversy [12, 13]. For this reason, non-invasive parameters such as urinary flow rate and residual urine measurement by ultrasound are utilised—this is widely available, easy to perform and are probably the most frequently used tests in urology today [14].

CHAMP is a longitudinal study of ageing in men, designed to prospectively measure a wide range of health issues in a contemporary Australian metropolitan population. Baseline data were collected in 2005 and participants have subsequently been seen at a number of time points. It is the first ever longitudinal study assessing the natural history of LUTS with symptom score as well as urinary flow rate and post-void residual (PVR) in a large cohort of community dwelling older men. The aim of this study was to assess the natural history of male LUTS in older men by utilising the data collected at baseline, 2 and 5 year follow-up.

## Materials and methods

### Study participants

The Concord Health and Ageing in Men Project (CHAMP) is a prospective cohort study of a wide range of health issues in older men. Baseline data were collected between January 2005 and June 2007. The study was approved by the Concord Hospital Human Research Ethics Committee. All participants gave written informed consent.

CHAMP involves 1705 men aged 70 years and over living in a defined region of metropolitan Sydney, Australia. The sampling frame was the New South Wales Electoral Roll, on which registration is compulsory. The only exclusion criterion was living in a residential aged care facility (nursing homes). The participation rate was 47%. The details of the sample selection process have been described previously [15].

### Measurements

Men were assessed at baseline, 2 and 5 years. Men completed a questionnaire at home before coming to the study clinic. This included questions on demographic characteristics, history of diagnosed medical conditions including, diabetes, stroke, Parkinson’s Disease, epilepsy, prostate cancer, LUTS using the IPSS [16] and history of surgery for benign prostatic hyperplasia (BPH).

Variables assessed at the interviews by trained personnel included the Mini-Mental State Examination [17] and functional disability (Katz activities of daily living [18]). Also, participants brought medications that they had been taking daily or almost daily for at least the past month to the clinic visit. Poor mobility was defined as needing help with walking across a small room and/or transferring from bed to a chair [18]. Use of urological medications (alpha blockers, five-alpha reductase inhibitors and urinary-specific antispasmodics) and diuretics was determined from the medication inventory.

At the clinic visit, urinary flow rate was performed using Urodyn 1000 (Medtronic Functional Diagnostics A/S, Skovlunde, Denmark) and PVR was measured using BladderScan BVI 3000 (VerathonInc, Bothell, WA, USA). All urinary flow rate graphs and BladderScan tracings were manually examined for artefacts by an urologist prior to entry into the CHAMP database. Outlying flow rates were further counter-reviewed by a second urologist and correlated with study records. Peak flow rate and PVR were analysed only when the voided volume was 150 ml and over.

The self-administered questionnaire and clinical assessments were repeated at 2 and 5 year follow-up visits. Telephone calls to participants were performed every 4 months.

### Analysis

Men who had prostate cancer at baseline or any follow-up points were excluded from analyses as this was likely to affect later management decisions. Having BPH surgery and use of urological medications during the 5-year follow-up period was recorded and accounted for. To estimate whether changes over time were statistically significant, a linear mixed model with an unstructured covariance matrix was used for each outcome measure with the least significant difference *post hoc* comparisons based on estimated marginal means. Correlations between IPSS, peak flow and residual volume and age were investigated using Spearman’s correlation coefficient. Statistical significance was considered at *P* < .05. All analyses were undertaken using SPSS version 24 (IBM, USA).

**Figure 1:**
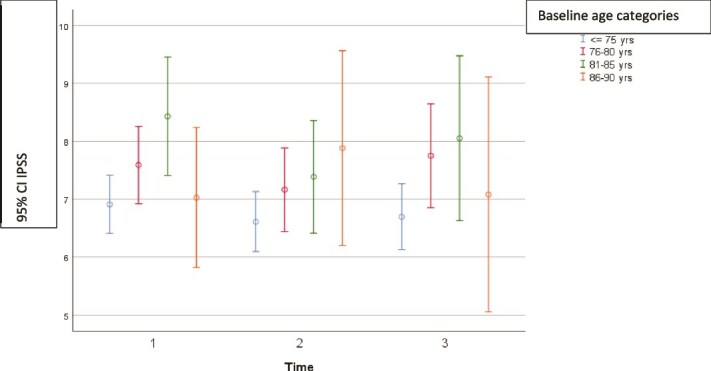
IPSS score at three timepoints (1 = baseline, 2 = 2 years and 3 = 5 years) by age at baseline. Mean, with a 95% confidence interval.

## Results

A total of 1705 men aged 70–97 years were recruited and subsequently participated in the study (the baseline CHAMP group). At 2-year follow-up, 1367 were seen (the 2-year CHAMP group). At the final 5-year assessment, a total of 940 men were seen (the 5-year CHAMP group). At 5 years, 382 men (22.4%) had died, 102 (6.0%) men declined follow-up and 186 (10.9%) men were too unwell for follow-up.

Participants residing within the CHAMP geographical area were born in Australia (50%), Italy (19%), the United Kingdom (4%), Greece (4%), China (3%) or other countries (20%). At baseline, 39% of men were aged 70–74 years, 31% 75–79 years, 19% 80–84 years, 8% 85–89 years and 3% were 90 years or older.

Prostate cancer was diagnosed in 275 (16.1%) of men either at baseline (214 men, 12.6%) or during the study period (61 men, 3.6%). Data for these men were excluded from all data analyses. Of these men, 33% underwent radiation treatment, 38% had surgery, 30% had androgen deprivation therapy alone or in combination with surgery or radiation and 9% had no treatment/observation only.

A clinical diagnosis of BPH had been made in 36.5% of men at study entry, usually by the General Practitioner. The 302 men who had undergone surgery for LUTS were excluded from analyses of natural history. Surgery for LUTS occurred in 65 (6.7%) men between baseline and 5 years and 6.0% were on prescription medication for LUTS. Out of 1705 men at baseline, 91 were on alpha-blockers, 12 on 5-alpha reductase inhibitors (5-ARI) and 30 on bladder-specific anticholinergics.

The mean IPSS were 7.35 at baseline, 6.96 at 2 years (*P* = .98 compared with baseline) and 7.18 at 5 years (*P* = .30 compared with baseline). Whilst this change was not significant, when age was included in the mixed model analysis the change over time was significant (p = 0.015) (see Figure 1). There was a significant correlation between IPSS score and reduced peak flow at baseline, 2 and 5 years (*P* = .002). IPSS score was correlated with increased residual volume at baseline (*P* < .01), 2 years (*P* = .01) and at 5 years (*P* < .001).

Flow rate and PVR volume data were analysed for those men who voided >150 ml. There were 1881 sets of data over the three time periods, 847 at baseline, 636 at 2 years and 398 at 5 years.

**Figure 2:**
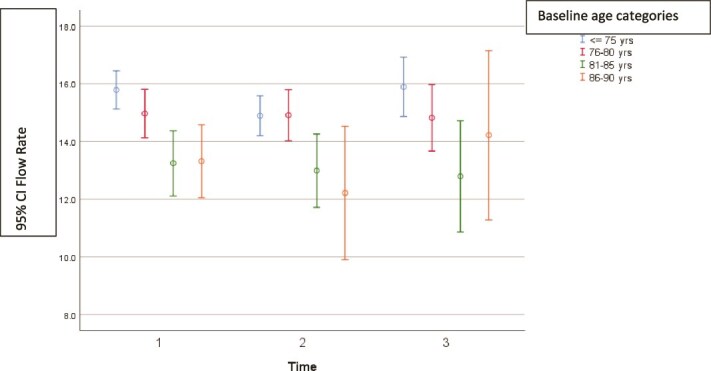
Peak flow rate at three timepoints (1 = baseline, 2 = 2 years and 3 = 5 years) by age at baseline. Mean, with a 95% confidence interval.

The mean peak flow rate at baseline was 15.0 ml/s, 14.6 ml/s at 2 years (*P* = .001 compared with baseline) and 15.3 ml/s at 5 years (*P* = .42 compared with baseline). The peak flow rate was generally lower in older men (81–85 years) compared to younger men (<75 years) both at baseline, 2 and 5 years (*P* = .002). Table 1 and Figure 2 shows the peak flow rate by age group over time. Adjusting for age at baseline, the change in peak flow over 5 years was not significant (*P* = .93).

**Table 1:** Peak flow in ml/s for each age group at baseline, 2 and 5 years, where voided volume was >150 ml. The *P* value compares baseline to 5 years (pairwise comparison).

	Baseline	2 years	5 years	*P* value
<75 years	15.74	14.80	15.39	0.34
76–80 years	15.05	14.64	14.71	0.54
81–85 years	13.31	12.93	13.29	0.99
86–90 years	13.34	12.17	12.59	0.76

**Figure 3:**
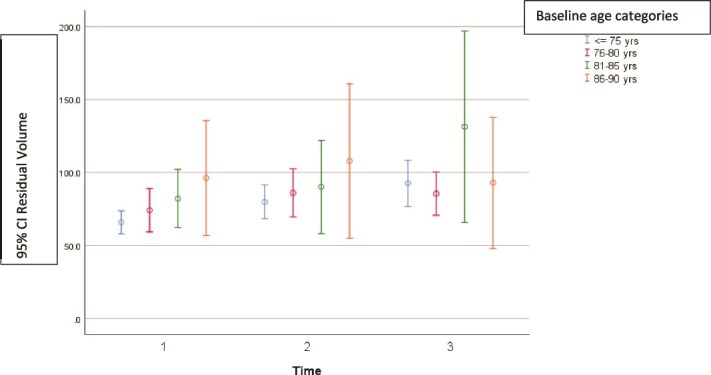
Post-void residual volume at three timepoints (1 = baseline, 2 = 2 years and 3 = 5 years) by age at baseline. Mean, with a 95% confidence interval.

Mean PVR was 72.4 ml at baseline, 84.0 ml at 2 years (*P* = .003 compared with baseline) and 93.2 ml at 5 years (*P* = .001 pairwise comparison). There was a trend to higher PVR in older men at each timepoint, although this was not significant (*P* = .068). Table 2 and Figure 3 shows the PVR by age group over time. The proportion of men with an elevated PVR of >100 ml were 23% at baseline, 25% at 2 years and 27% at 5 years. Men with an elevated PVR of >200 ml were 6% at all three time points. Men with an elevated PVR of >300 ml were 3% at baseline, 3% at 2 years and 4% at 5 years. In men with residual volume >200 ml at baseline the residual volume did not change significantly over 5 years (*P* = .51). Five of 31 men (16%) with PVR >300 ml at baseline had undergone transurethral resection of the prostate (TURP) by 5 years. Three of 34 (9%) men with PVR 200 to 300 ml had a TURP by 5 years. Ten men (0.08%) were using a catheter at baseline and 9 (0.07%) at 5 years.

**Table 2:** Post-void residual volume in ml for each age group, at baseline, 2 and 5 years, where voided volume was >150 ml. The *P* value compares baseline to 5 years.

	Baseline	2 years	5 years	*P* value
<75 years	65.4	76.7	88.0	00.001
76–80 years	73.1	84.8	91.6	0.056
81–85 years	77.5	91.6	116.7	0.039
86–90 years	99.8	101.3	107.3	0.861

## Discussion

LUTS are a common problem in older men. This is the first study to longitudinally assess the natural history of LUTS with symptom score (IPSS), flow rate and PVR volume in a population of community-dwelling older men over a period of 5 years. We have shown that IPSS remains stable with time, peak flow decreases with age and PVR increases over time. However, the magnitude of these changes is small and not likely to be clinically significant for many older men. The majority of older men had good quality of life from a urinary tract point of view, reporting only mild LUTS on IPSS and this proportion did not change with time. The number of men undergoing surgery was low.

A small proportion of men in our cohort had an elevation of PVR at baseline, i.e. commonly considered clinically significant (3% > 300 ml and 6% > 200 ml) but these cohorts had stable PVR throughout follow-up and the rates of men requiring surgical intervention were low. An in-depth analysis of the natural history of men in the CHAMP study with elevated PVR was reported by Noguchi et al. and confirmed the stability of the majority of men with elevated PVR < 300 ml [19]. In particular, we have found that men with an elevated PVR are not on a trajectory towards increasingly high PVRs and there is no evidence of clinical deterioration based on elevated baseline PVR.

Our results are consistent with previously published studies on the relationship between LUTS due to BPH as measured by IPSS and objective non-invasive parameters of lower urinary tract dysfunction. A weak correlation between IPSS, PVR and peak flow has previously been found. Furthermore, in older men, symptoms and IPSS may not be secondary to outlet obstruction but there is likely to be a contribution from bladder dysfunction, such as decreased bladder contractility [14].

BLSA followed 1057 men for up to 30 years with symptom questionnaires and digital rectal examinations to assess the prevalence of BPH in its population [8]. It found that the age-specific prevalence of clinically diagnosed BPH was consistent with the age-specific prevalence of autopsy defined BPH from independent autopsy studies. Furthermore, it found that the 20-year risk of surgery for BPH was 39% for men 60 years of age and older.

The Olmstead County Study followed 2115 men from 40 to 79 years for 6 years with a 55% response rate [9]. They used a symptom questionnaire, urinary flow rate, PSA and a random 25% transrectal ultrasound to assess prostate volume. In follow-up, 167 men required medical or surgical treatment for BPH. Factors associated with requiring treatment were a depressed peak flow rate (<12 ml/s), moderate to severe symptoms (IPSS > 7) and an enlarged prostate (>30 ml). Almost 25% of men aged 70–79 years required treatment. The rate of intervention is higher than our findings but may be explained by different patterns of care between the USA and Australia and the fact that 18% of the total CHAMP cohort having received surgical treatment for BPH at entry to the study were excluded from the current analysis.

A Japanese study [20, 21] followed 289 men for 3 years from 40 to 79 years, with 77% follow-up. IPSS, peak flow and prostate volume were measured at baseline. None of these measures predicted IPSS progression but men with IPSS scores in the severe category were more likely to undergo TURP (21%).

In the CHAMP cohort, there was a trend towards minor deterioration in peak flow rates with age, which are not statistically significant. Men aged 86–90 years had a reduction in peak flow rate from 13.34 ml/s to 12.59 ml/s over 5 years and men <75 years from 15.74 ml/s to 15.39 ml/s. It is therefore reasonable to conclude that routine follow-up of patients with elevated PVR or reduced peak flow is not necessary in the majority of older men. Assessment and follow-up can be appropriately based on self-reported symptoms and/or IPSS.

The strengths of our study are that the CHAMP data set is a robust, longitudinal study of older men with 5 year follow-up and very low rates of loss to follow-up (6%) compared to other major longitudinal published data. Both subjective data and objective urinary flow rate data were obtained and recorded for all patients, and all urinary flow rate and PVR data were manually checked by an urologist prior to acceptance into the study database. Furthermore, this is a unique cohort of men to study the natural history of LUTS as only 7% were on medical therapy and very few (*n* = 12) were on 5-ARI due to the lack of reimbursement for 5-ARI in Australia during the time of the study. The data really suggests that quality of life is good with conservative treatment and the risk of intervention is low. Study limitations include the fact that the CHAMP study excludes men living in residential care facilities, and the likelihood of the ‘survivor’ effect in the relatively small numbers of the oldest participants (age 86–90) in our cohort. The survivor bias may also contribute to the characteristics of the remaining cohort at 5 years, if less healthy men who were unable to continue in the study had deterioration in LUTS. This analysis has also excluded men with prostate cancer, so caution should be taken when generalising our findings to this cohort.

## Conclusions

Urinary symptoms and voiding parameters remain remarkably stable over 5 years in community dwelling older males. Men with elevated PVR urine did not deteriorate significantly and the number of men requiring surgical intervention was low. Conservative management of LUTS appears to be a reasonable strategy for the majority of older men.
